# Evaluation of anterior segment parameters between pregnancy trimesters and postpartum with pentacam scheimflug ımaging: a prospective study

**DOI:** 10.1007/s10792-024-03173-y

**Published:** 2024-06-24

**Authors:** Çisil Erkan Pota, Aslı Çetinkaya Yaprak

**Affiliations:** 1 Department of Ophthalmology, Antalya Manavgat State Hospital, Manavgat, Antalya Turkey; 2 Department of Ophthalmology, Akdeniz University Faculty of Medicine, 07070 PınarbaşıMah., Konyaaltı, Antalya Turkey

**Keywords:** Anterior chamber angle, Anterior chamber depth, Corneal thickness, Corneal volume, Pregnancy

## Abstract

**Purpose:**

To evaluate the effect of pregnancy on the anterior chamber, corneal parameter, and intraocular pressure measurements; and compare the results between trimesters, postpartum and non-pregnant healthy age-matched women.

**Methods:**

This prospective study included 41 pregnant women and 53 non-pregnant women. Four measurements were taken from the pregnant women, in each trimester and postpartum third month, and once from the control group. Of the individuals included in the study, anterior chamber depth (ACD), anterior chamber volume (ACV), K1 (flat keratometry), K2 (steep keratometry), Kmean (mean value of K1 and K2), anterior chamber angle (ACA), central corneal thickness (CCT), thinnest corneal thickness (TCT), astigmatism value (AST), corneal volume (CV), biometry, axial length (AL), spherical equivalent (SFEQ), intraocular lens power (ILP), VA (visual acuity) datas were recorded.

**Results:**

We observed a statistically significant decrease in K2, CCT, ACD, AL and CV in the postpartum period (*p* = 0.025, *p* < 0.001, *p* = 0.029, *p* = 0.005, *p* = 0.004 respectively) and a statistically significant increase in ACV, CCT, and TCT as the gestational week progressed in the pregnant group (*p* = 0.007, *p* < 0.001, *p* = 0.025, respectively). A statistically significant decrease in IOP towards to the third trimester, and an increase in the postpartum period was observed (*p* < 0.001). We did not observe statistically significant changes in K1, Kmean, AST, ACA, VA, ILP, and SFEQ values.

**Conclusion:**

It is important to investigate the physiological changes that may occur during pregnancy, distinguish them from pathological changes, and avoid unnecessary treatment. We consider that it’s also important to guide the timing of anterior segment surgeries such as cataract and refractive surgery and to prescribe glasses/contact lenses.

## Introduction

Pregnancy is a physiological process that affects many systems such as cardiovascular, pulmonary, renal, hematological, and visual systems, especially the endocrine system [1]. Hormonal, coagulative, and hemodynamic changes are responsible for most of the ocular adaptations [2]. In previous studies, changes between trimesters were observed in anterior segment parameters in pregnant women. These changes are a decrease in intraocular pressure (IOP) [3], a decrease in corneal sensitivity [4], an increase in corneal curvature [5] and a decrease in central corneal thickness [6]. Although the etiology is not known, it is returned to first trimester values at the end of the breastfeeding period; therefore, it has been suggested that it may occur due to corneal edema due to hormonal changes [7].

Anterior segment topographies are currently used to evaluate anterior segment parameters. Using Pentecam Scheimplug technology (Pentecam Oculus Optikgeräte GmbH, Wetzlar, Germany) gives us detailed information about anterior segment parameters [8]. Since it is a noncontact and noninvasive method, it is extremely safe to use in special patient groups such as pregnant women.

There is a limited number of studies evaluating anterior segment parameters in pregnant women. In these studies, measurements of different pregnant subjects were compared while evaluating the measurements between trimesters [9, 10]. We think that the anterior segment parameter values obtained from different individuals may affect the results of the studies due to individual differences [10]. Measurements taken from the same individuals increase the reliability of the results, and there is no study in the literature in which the same individuals are followed in each trimester of pregnancy. Therefore, in this study, we measured a total of 4 measurements, in the first, second, third, and postpartum periods, from subjects who were followed up from the beginning of pregnancy and compared them with the healthy age- and sex-matched control group.

## Material method

This prospective case–control study was approved by the local ethics committee of the Akdeniz University Faculty of Medicine (Approval Number KAEK-841) and the study was conducted in compliance with the ethical standards set out in the Declaration of Helsinki. Informed consent was obtained from the participants who agreed to participate in the study.

All subjects underwent a comprehensive ophthalmological examination, including measurements of refractive error (Nidek ARK-700A, Nidek Co., Ltd, Gamagori, Japan), best-corrected visual acuity (BCVA), IOP /Full Auto Tonometer, NIDEK NT-2000, Nidek Co., Ltd., Aichi, Japan), biometry and axial length (AL), (IOLMASTER 500, Carl Zeiss Meditec AG, Jena, Germany), slit-lamp examination of the anterior and posterior segment, and Scheimflug Topography (Pentecam Oculus Optikgeräte GmbH, Wetzlar, Germany). The BCVA was converted into the logarithm of minimal angle resolution (logMAR).

Of the individuals included in the study, anterior chamber depth (ACD), anterior chamber volume (ACV), K1 (flat keratometry), K2 (steep keratometry), Kmean (mean value of K1 and K2), anterior chamber angle (ACA), central corneal thickness (CCT), thinnest corneal thickness (TCT), astigmatism value (AST), corneal volume (CV), axial length (AL), spherical equivalent (SFEQ), intraocular lens power (ILP), and VA (visual acuity) data were recorded.

Pregnant women were followed for about 1 year, in the first, second, third trimesters, and postpartum third months, and four measurements were taken from the same pregnant subjects in total. One measurement was taken from the control group. All measurements were made by the same ophthalmologist (ÇEP) between 14:00 and 16:00 to avoid diurnal variation. All participants had no pathology in their right eyes, and the right eyes of the participants were included in the study. The study began with 55 patients from both groups. 14 patients from the pregnant group were excluded from the study because they did not continue the follow-up, and 2 patients from the control group were excluded because their image quality was not good.

*Inclusion criteria for the pregnant group* Uncompleted singleton pregnancies, who are at a gestational age less than 14 weeks in the first examination and continue their follow-up, and who do not have a systemic disease related to pregnancy (gestational diabetes, preeclampsia/eclampsia, etc.).

*Inclusion criteria for the control group *Nonpregnant healthy women not in menopause and not taking hormone replacement therapy.

*Inclusion criteria for both groups* Between 20 and 40 years of age, without any systemic disease (such as diabetes mellitus, hypertension, thyroid disease), not using systemic and/or ocular drugs, with a spherical refractive error of less than 4 diopters (D) and/or cylindrical refractive errors of less than 2 D. Individuals without previous ocular surgery and ocular pathology affecting visual acuity and anterior segment parameters were included in the study.

## Statistical analysis

Statistical analyzes were performed using the IBM SPSS package, version 23.0 (SPSS Inc., Chicago, IL, USA). The Shapiro–Wilk test was used to analyze the normality of sample distribution. To define the sample, normally distributed values are presented as means ± standard deviation, and non-normally distributed values are presented as median (minimum–maximum). The two-way repeated measures ANOVA test was used to examine the time-dependent change of the data. For the analysis of independent data, the independent-sample t-test and Mann–Whitney U test were used. For the analysis of correlation, Spearman’s correlation coefficient was used. The results were evaluated at a 95% CI. A level of *p* < 0.05 was accepted as statistically significant.

## Results

A total of 94 eyes, including 41 pregnant women and 53 eyes of non-pregnant healthy age- and sex-matched controls, were included in the study. The demographic features of the subjects are summarized in Table 1. No statistically significant difference was observed between the mean ages of the subjects in the groups. (*p* = 0.217) (Table 1). Mean values of all anterior segment parameters in control, first, second, and third trimesters and postpartum groups and *p* values are shown in Table 1 and Table 2.

**Table 1:** Demographical values and mean values of anterior segment parameters (mean ± SD (standart deviation))

Variable	Control (n = 53)	First trimester (n = 41)	Second trimester (n = 41)	Third trimester (n = 41)	Postpartum (n = 41)	*p* (control-pregnant group)	*P* (between trimesters of pregnancy)	*P* (between pregnant and postpartum measures)
Age (years) Min–Max	30.8 ± 5.8	29.2 ± 6.4	–	–	–	0.218		
	20–40	20–40						
Weeks of pregnancy/postpartum Min–Max	–	9.5 ± 2.7	20.1 ± 2.4	31 ± 2	12 ± 1			
		5–13	17–27	29–36	10–14			
IOP (mmHg)	15.78 ± 0.35	14.74 ± 0.49	13.95 ± 0.47	12.93 ± 0.4	14.64 ± 0.46	**0.002**	**< 0.001**	**< 0.001**
SFEQ	− 0.932 ± 0.209	− 0.870 ± 0.18	− 0.841 ± 0.185	− 1 ± 0.216	− 0.905 ± 0.214	0.775	0.192	0.199
ILP (diopters)	20.26 ± 0.44	20.98 ± 0.35	20.7 ± 0.4	20.86 ± 0.35	20.9 ± 0.41	0.204	0.209	0.056
AL (mm)	23.86 ± 0.94	23.36 ± 0.72	23.36 ± 0.74	23.39 ± 0.76	23.38 ± 0.16	**0.027**	0.087	**0.005**
VA (logMAR)	0.0047 ± 0.003	0.0073 ± 0.003	0.0085 ± 0.003	0.0073 ± 0.003	0.0073 ± 0.003	0.273	0.496	0.528

Statistically significant *p* values are indicated in bold

*Min* minimum, *Max* maximum, *IOP* intraocular pressure, *SFEQ* spherical equivalent, *ILP* intraocular lens power, *AL* axial length, *VA* visual acuity values are expressed as mean ± SD. In control-pregnant group comparisons, all measurements taken from the pregnant woman and the measurements of the control group were compared

**Table 2:** Mean values of anterior segment parameters (mean ± SD (standart deviation)) in control, first, second and third trimesters and postpartum groups and *p* values

Variable	Control	First trimester (n = 41)	Second trimester (n = 41)	Third trimester (n = 41)	Postpartum (n = 41)	*P* (control-pregnant group)	*P* (between trimesters of pregnancy)	*P* (between pregnant and postpartum measures)
K1 (diopters)	42.85 ± 1.44	43.31 ± 1.45	43.41 ± 1.46	43.46 ± 1.61	43.35 ± 1.43	0.301	0.099	0.074
K2 (diopters)	44.22 ± 1.64	44.57 ± 1.82	44.52 ± 1.81	44.44 ± 1.66	44.49 ± 1.79	0.353	0.137	**0.025**
Kmean (diopters)	43.37 ± 1.49	43.67 ± 1.5	43.49 ± 1.72	43.79 ± 1.42	43.91 ± 1.53	0.513	0.898	0.347
AST (diopters)	1.01 ± 0.52	1.03 ± 0.72	0.94 ± 0.74	0.84 ± 0.58	1.22 ± 0.8	0.978	0.066	0.111
CCT (µ)	546.16 ± 46.03	542.23 ± 27.47	540.41 ± 26.47	546.58 ± 28.88	544.58 ± 29.44	0.742	**< 0.001**	**< 0.001**
TCT (µ)	541.97 ± 46.44	541.46 ± 28.80	540.30 ± 29.91	544 ± 27.79	537.43 ± 36.12	0.976	**0.025**	0.063
ACV (mm^3^)	177.97 ± 28.46	165.9 ± 26.73	168.2 ± 27.3	171.5 ± 31.5	164 ± 29.4	**0.002**	**0.007**	0.161
ACD (mm)	3.05 ± 0.29	2.93 ± 0.25	2.97 ± 0.32	2.91 ± 0.28	2.91 ± 0.27	0.141	**0.024**	**0.029**
ACA (º)	38.87 ± 6.42	37.8 ± 4.74	37.32 ± 4.08	37.1 ± 3.8	37.69 ± 4.29	0.475	0.291	0.251
CV (mm^3^)	61.16 ± 3.4	58.5 ± 3.26	58.22 ± 3.34	58.37 ± 3.23	57.92 ± 3.26	0.242	0.122	**0.004**

Statistically significant *p* values are indicated in bold

*K1* flat keratometry, *K2* steep keratometry, *Kmean* mean value of K1 and K2, *AST* astigmatism, *CCT* central corneal thickness, *TCT* thinnest corneal thickness, *ACV* anterior chamber volume, *ACD* anterior chamber depth, *ACA* anterior chamber angle, *CV* corneal volume, *IOP* intraocular pressure, *ILP* intraocular lens power, *SFEQ* spherical equivalent, *AL* axial length, *VA* visual acuity values are expressed as mean ± SD. In control-pregnant group comparisons, all measurements taken from the pregnant woman and the measurements of the control group were compared

There was a statistically significant difference between trimesters and K1 values in the pregnancy group; there was no statistically significant difference between the postpartum period and the control group (*p* = 0.076, *p* = 0.301, respectively). We found a statistically significant increase in K2 value in the second trimester compared to the first trimester in the pregnancy group (*p* = 0.038), but we did not observe a statistically significant difference when the three trimesters were evaluated (*p* = 0.137). We found the K2 values during pregnancy to be statistically significantly higher than the postpartum values (*p* = 0.025). We did not find a statistically significant difference in corneal astigmatism values between trimesters and when compared with the postpartum and control groups (*p* = 0.066, *p* = 0.111, *p* = 0.978, respectively) (Table 2).

**Fig. 1:**
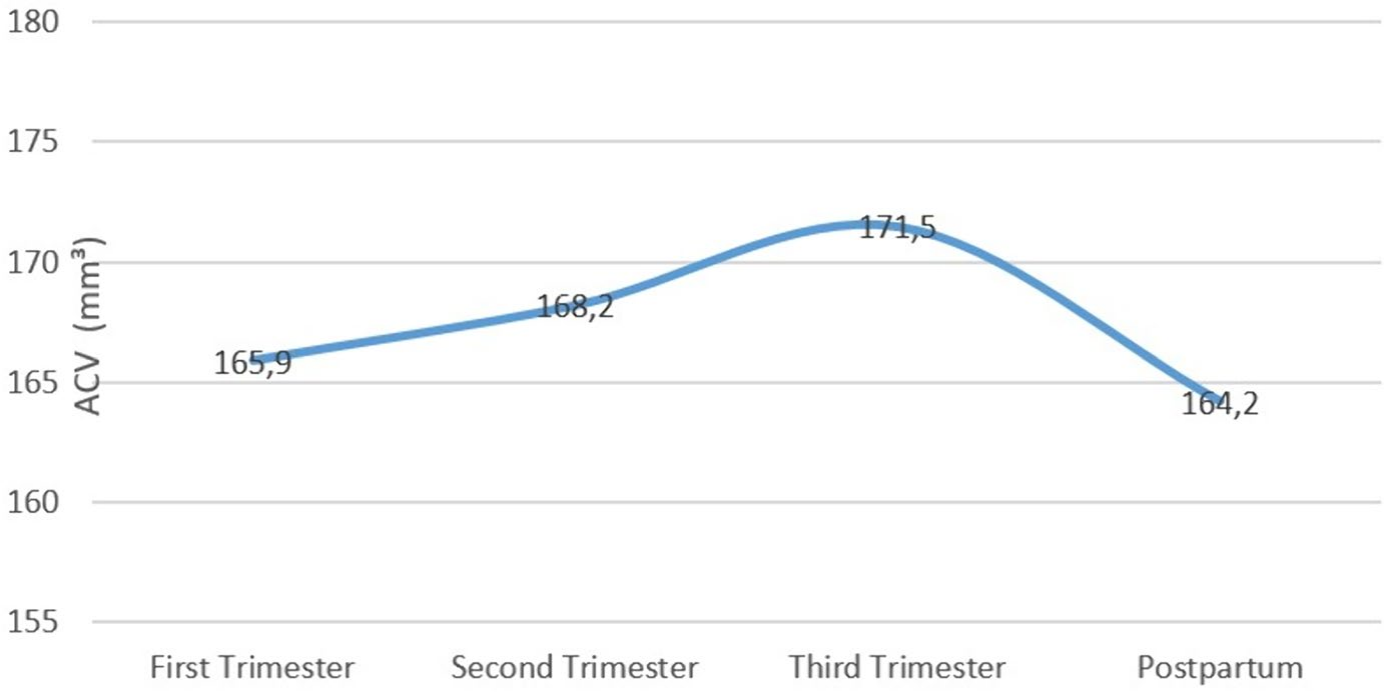
Anterior chamber volume changes during pregnancy and the postpartum period

When trimesters were compared, we observed a statistically significant increase in TCT and CCT values between trimesters in the pregnant subjects (*p* = 0.025, *p* < 0.001, respectively). We observed a statistically significant increase in ACV values as the gestational week progressed (*p* = 0.007) (Table 2). ACV values are shown in Fig. 1.

**Fig. 2:**
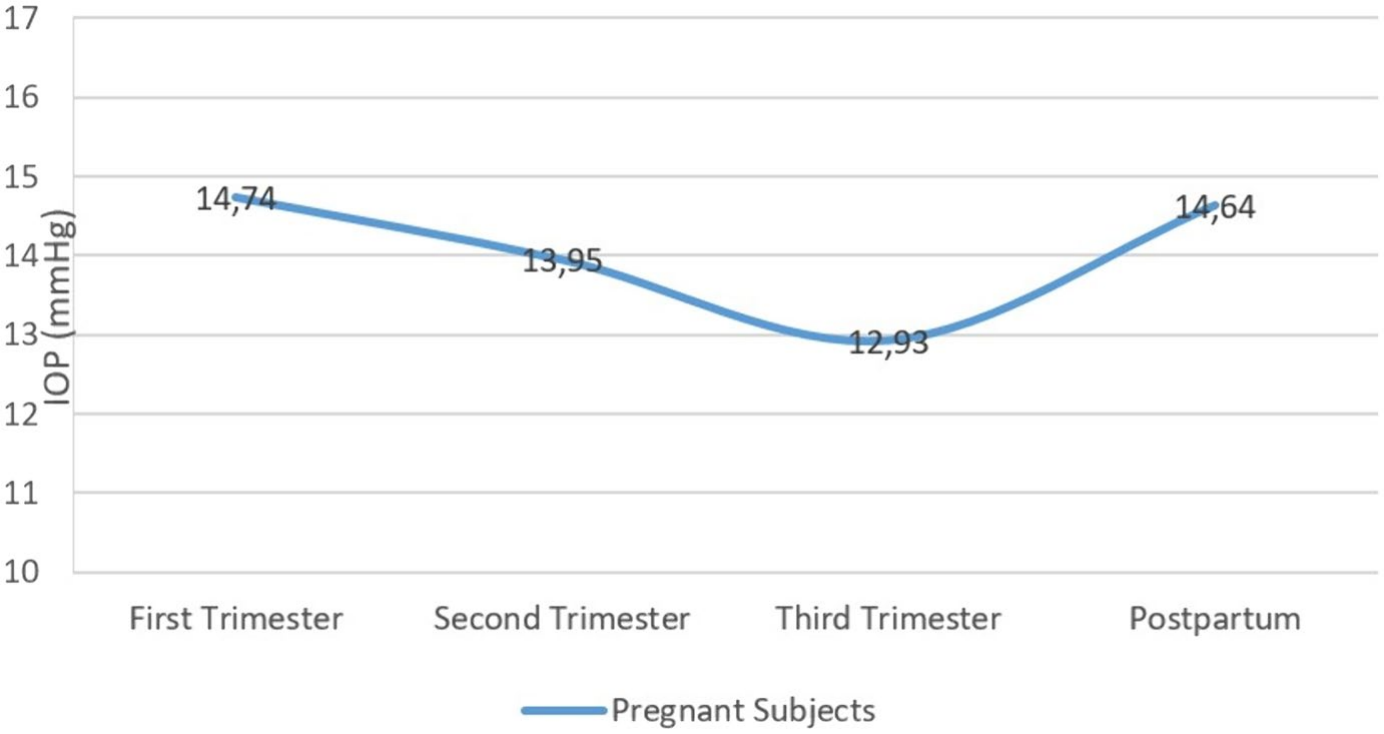
Intraocular pressure changes during pregnancy and the postpartum period

We found the CV measurements to be higher in the pregnancy group than the postpartum measurements (*p* = 0.004). When the trimesters were compared among themselves, we found a statistically significant increase in the 3rd trimester compared to the 2nd trimester (*p* = 0.025). There was a significant decrease in IOP between trimesters and compared to the postpartum period and control group (*p* < 0.001, *p* < 0.001, *p* = 0.002, respectively) (Table 1). The IOP change is shown in Fig. 2.

When we examined the correlation between the gestational week and the measured values, there was a negative correlation between IOP and gestational week (*p* = 0.002, r =  − 0.275). We observed a positive correlation between gestational week in ACD (*p* = 0.008, r = 0.299). We did not find a statistically significant correlation between other parameters and gestational week.

## Discussion

In our study, we found a statistically significant decrease in K2, CCT, ACD, AL and CV in the postpartum period and a statistically significant increase in ACV, CCT, and TCT as the gestational week progressed in the pregnant group. We found a statistically significant decrease in IOP towards to the third trimester, and an increase in the postpartum period. We did not find statistically significant changes in K1, Kmean, AST, ACA, VA, ILP, and SFEQ values.

During pregnancy, many organs, including the eye and orbit, are affected by the physiological interaction between the mother and the fetus. These changes are more prominent, especially in the third trimester and regress in the postpartum period [11–13]. It was thought that the steepening of the corneal curvature would increase the refractive power of the cornea, but this effect would be slightly compensated due to the existing corneal edema [7, 14]. In our study, we found that myopia increased towards the last trimester and decreased in the postpartum period. This is in agreement with Pizzarello’s study, they found a change in favor of myopia during pregnancy and it returned to pre-pregnancy in the postpartum period [15]. This is in contrary to Ataş et al. refractive values were similar during the third trimester and the postpartum period [13].

In our study, we found that AL increased during pregnancy, but this increase was not statistically significant. However, in pairwise comparisons, it increased in the third trimester compared to the first trimester (*p* = 0.042) and decreases in postpartum measurements. This is in agreement with Sen et al. they evaluated the same parameters with the same device (IOL Master 500 Carl Zeiss Meditec Inc.. Jena. Germany) [16].

In our study, we did not observe a statistically significant difference between biometry values and intraocular lens power measurement during pregnancy and in the postpartum period. There was no difference between the control group and the pregnant group, and we did not observe any significant changes in visual acuity during pregnancy. This is in agreement with Manchester and Taner et al. no accommodative disorders or visual impaierment were found in the examination of pregnant women [7, 17].

In our study, we found that the K1 value increased towards the third trimester, however, the difference was not statistically significant (*p* = 0.09). Furthermore, there was no statistically significant change in K2 value during pregnancy. This is in agreement with Efe, Manchester and Taradaj et al. observed no significant alterations in keratometric parameters due to pregnancy [6, 10, 12]. This is in contrary to Park. and Manchester et el. observed an increase in corneal curvature during the second and third trimesters which resolved postpartum or after the cessation of breastfeeding [5, 7]. Although the etiology of this steepening of the cornea is not clear, it has been suggested that it may occur due to corneal edema caused by hormonal changes [7]. Based on this information, it was recommended not to prescribe glasses and contact lenses to pregnant women because of variable keratometry [7]. We think that the differences in K values in previous studies might be due to the comparison of different pregnant subjects for each trimester.

Many studies have been conducted on IOP changes during pregnancy [18, 19]. Most studies found a decrease in IOP during pregnancy [20]. The mechanism of IOP decrease was not exactly clarified. It is considered that increased levels of estrogen, relaxin, progesterone, and β-human chorionic gonadotropin amplify the outflow of aqueous humor through the unconventional track. [6, 12, 13, 21]. Since the reduction in systemic vascular resistance causes a reduction in episcleral venous pressure causes a reduction in IOP is another possible factor [18]. We found a 12% IOP decrease in third trimester of pregnancy and intraocular pressure returned to first trimester values in the postpartum period and the decrease in intraocular pressure increased as the gestational week progressed. This is in agreement with Tolunay, Özkaya, Efe, and Weinreb et al. who reported that IOP decreased in the second and the third trimesters compared to the first trimester [6, 10, 22, 23].

There are limited studies in the literature evaluating ACV, ACD, and ACA measurements in pregnancies. Changes in anterior chamber parameters are expected due to increased aqueous humor outflow and fluid retention in the body during pregnancy [13]. In our study, we observed a statistically significant increase in ACV in the second trimester and a decrease in the postpartum period. We did not detect any changes in ACA values. This is in agreement with Ataş et al. ACV values were significantly higher during the third trimester compared with the third month postpartum [13]. This is in agreement with Goldich et al. they observed no significant differences in the ACD, or ACA between pregnant women in their third trimester and nonpregnant women [21]. However in contrary to our study, Goldich et al. did not detect any difference in ACV values [21]. To the best of our knowledge, our study is the first study in which these parameters were evaluated prospectively during pregnancy trimesters and the postpartum period. Further study series with larger numbers of patients are needed to further comment on changes in the anterior chamber.

Weinreb et al. suggested that fluid retention during pregnancy can lead to higher corneal thickness [23]. This situation was previously explained by the inclusion of androgen, estrogen, and progesterone receptors in the human cornea [11, 24, 25]. In our study, we found a significant increase in CCT in the third trimester, and we found the corneal thickness decreased in the postpartum period. This is in agreement with Efe et al. the mean CCT in the second and third trimesters of pregnancy was measured to be higher than in the first trimester and at 3 months postpartum period [6]. Similarly, this is in agreement with Atas et al. demonstrated an increase in corneal thickness in the third trimester of pregnancy compared to the postpartum period [13]. The results of prospective studies were similar to our study. Errors that may arise from individual differences could be eliminated with measurements taken from the same individuals [26].

In addition to corneal thickness, several previous studies have evaluated corneal volume (CV). In our study although the increase in corneal volume was detected in the last trimester was not statistically significant; the decrease in the postpartum period was statistically significant (*p* = 0.04). We observed the corneal volume to be significantly higher starting from the first trimester compared to the postpartum period. This could show that the increase in corneal volume begins in the early stages of pregnancy and returns to normal after delivery. This is in contrary to Goldich et al. no difference was found between the third trimester pregnant and control groups [21]. This is in agreement with Ataş et el. CV was higher during the third trimester of pregnancy in comparison with the third month postpartum [13].

This study has some limitations. First, the sample size is small. Second, although the menstrual cycle can affect corneal biomechanical parameters we couldn’t take measurements from non-pregnant and postpartum women at the same menstrual cycle stage. Another limitation is that no measurements were made after the cessation of breastfeeding. Larger studies with larger sample groups are needed.

In conclusion, we found a statistically significant increase in ACV, CCT, and TCT towards the third trimester in the pregnant group, and a statistically significant decrease in K2, CCT, ACD, AL and CV in the postpartum period. We found a statistically significant decrease in IOP towards the third trimester and a statistically significant increase in the postpartum period. We think that it is important to investigate the physiological changes that may occur during pregnancy, to distinguish it from pathological changes, to avoid unnecessary treatment, to prescribe glasses/contact lenses, and to guide the timing of anterior segment surgeries such as cataract and refractive surgery.

## Data Availability

All data and materials are available from the supplementary material.
